# Surface-enhanced Raman spectroscopy of tears: toward a diagnostic tool for neurodegenerative disease identification

**DOI:** 10.1117/1.JBO.25.8.087002

**Published:** 2020-08-06

**Authors:** Gilda Cennamo, Daniela Montorio, Vincenzo Brescia Morra, Chiara Criscuolo, Roberta Lanzillo, Elena Salvatore, Carlo Camerlingo, Mikhail Lisitskiy, Ines Delfino, Marianna Portaccio, Maria Lepore

**Affiliations:** a Universitá “Federico II” di Napoli, Dipartimento di Sanitá Pubblica, Napoli, Italy; b Universitá “Federico II” di Napoli, Dipartimento di Neuroscienze e Sci. Riproduttive e Odontostomatologiche, Napoli, Italy; c CNR-SPIN, Ist. Superconduttori, Materiali Innovativi e Dispositivi, Consiglio Nazionale delle Ricerche, Pozzuoli, Italy; d Universitá della Tuscia, Dipartimento di Scienze Ecologiche e Biologiche, Viterbo, Italy; e Universitá della Campania “L. Vanvitelli,” Dipartimento di Medicina Sperimentale, Napoli, Italy

**Keywords:** neurodegenerative diseases, surface-enhanced Raman spectroscopy, Alzheimer’s disease, i-PCA analysis, tears

## Abstract

**Significance:** A noninvasive method based on surface-enhanced Raman spectroscopy (SERS) of tears was proposed as a support for diagnosing neurodegenerative pathologies, including different forms of dementia and Alzheimer’s disease (AD). In this field, timely and reliable discrimination and diagnosis are critical aspects for choosing a valid medical therapy, and new methods are highly required.

**Aim:** The aim is to evince spectral differences in SERS response of human tears from AD affected, mild cognitive impaired (MCI), and healthy control (Ctr) subjects.

**Approach:** Human tears were characterized by SERS coupled with multivariate data analysis. Thirty-one informed subjects (Ctr, MCI, and AD) were considered.

**Results:** Average SERS spectra from Ctr, MCI, and AD subjects evidenced differences related to lactoferrin and lysozyme protein components. Quantitative changes were also observed by determining the intensity ratio between selected bands. We also constructed a classification model that discriminated among AD, MCI, and Ctr subjects. The model was built using the scores obtained by performing principal component analysis on specific spectral regions (i-PCA).

**Conclusions:** The results are very encouraging with interesting perspectives for medical applications as support of clinical diagnosis and discrimination of AD from other forms of dementia.

## 1 Introduction

Neurodegenerative diseases, particularly affecting aged people, have relevant health and social costs. Different forms of diseases, often involving serious forms of dementia, are included in this class of pathology requiring specific clinical treatments to be fronted. Thus, timely and reliable diagnosis is a critical aspect for choosing a valid medical therapy. A mild cognitive impairment (MCI) reflects a cognitive decline but not yet a state of dementia and often does not compromise daily life activities.^1^ Alzheimer’s disease (AD) is a more severe neurodegenerative pathology characterized by a progressive damage of synaptic links and neuronal cells mainly in the hippocampus brain region. The occurrence of pathological aggregates of proteins including beta-amyloid fragments (Aβ-plaques) in the interneuronal spaces of brain tissue and the increased level of tau- and hyperphosphorylated-tau proteins in the cerebrospinal fluid of AD-affected subjects indicated the relevance of beta-amyloids (Aβ) and tau-protein in the AD development.^2^^–^^4^ Both substances are biomarkers of AD progression^2^^,^^4^ and are a reliable sign of a neurodegenerative cerebral process also in the early-stage phase.^5^^–^^7^ These biomarkers can be transported into the vascular system across the blood–brain barrier (BBB).^8^^,^^9^ The BBB plays an important role in the selective passage of several substances between brain and blood systems and in the maintenance and integrity of the brain. A dysfunctional BBB with impaired ability to clear Aβ and tau protein from the brain has been associated with the development of AD.^10^ Traces of these biomarkers are present in the systemic blood circulation and can rejoin the vascularization of other body districts, as the retina, lens, and lacrimal regions, affecting the composition of tears.^11^^–^^13^ The relevance of changes occurring in the eye as a consequence of AD has been reported by Lim et al.^14^ Consequently, tears are an ideal medium for investigating changes induced by neurodegenerative pathologies. They are easily accessible and can be collected using minimally invasive methods.

In the last decades, a large number of studies have been addressed to analyze the tear composition by using conventional biochemical methods, as high-performance liquid chromatography, enzyme-linked immunosorbent assays, or ferric reducing antioxidant power, and have allowed an exhaustive characterization of this fluid.^15^^–^^17^ These conventional techniques have been largely superseded by optical spectroscopy methods, such as those based on Raman scattering, that are less time consuming, do not require specific preliminary preparation of the samples, and have good sensitivity and specificity. Raman spectroscopy has been used to identify AD biomarkers in blood serum^8^^,^^18^^,^^19^ and saliva,^20^ and to investigate basic aggregation mechanisms of amyloids.^21^ Raman spectroscopy^22^^–^^25^ and surface-enhanced Raman spectroscopy (SERS)^26^ have also been used to investigate human tears mainly to diagnose eye diseases. In addition to the direct detection of biomarkers, Raman and SERS can evaluate the global changes that the pathological state induces in the tear composition. A relevant change in the protein profiles between tear samples collected from AD-affected and healthy subjects was observed by Kalló et al.^27^ using electrophoresis analysis (Bradford method). Filik and Stone^28^ demonstrated that Raman spectroscopy can be used to analyze tear composition. However, some limits concerning sensitivity, noise/signal level, and repeatability also related to the inhomogeneous composition of samples can be evinced.

The development of SERS overcame some limits of conventional Raman spectroscopy. Indeed, it dramatically increased the sensitivity and opened new perspectives in fluid spectroscopic analysis.^29^^–^^31^ For instance, Ryzhikova et al.^31^ highlighted a significant improvement in human serum analysis for AD diagnosis. We previously investigated the potential of SERS in the study of human tears.^26^ Tear samples were analyzed using a gold-nanoparticle-based substrate whose SERS enhancement factor was evaluated by employing water solutions of rhodamine 6G. By comparing SERS signal with the one obtained by conventional Raman spectroscopy, an SERS enhancement factor of about $4\times 10^{3}$ was estimated. A direct comparison of SERS and Raman response of human tears has been also reported, clearly indicating a signal intensity improvement.^26^ In this work, we investigated human tear samples collected from informed subjects with ascertained AD and MCI symptoms using SERS. Spectroscopy results were compared with a reference signal obtained from healthy subjects. Principal component analysis (PCA) and interval principal component analysis (i-PCA) were used to efficiently and reliably discriminate spectra, with promising issues for the development of large-scale screening methods for diagnosis and discrimination of neurodegenerative diseases.

## 2 Materials and Methods

### 2.1 Participants

Eighteen AD affected subjects (7 women, 11 men, and mean age 71±10 years) and seven MCI subjects (3 women, 4 men, and mean age 73±9 years) were included in this study. All met the core clinical criteria for AD and MCI of the National Institute on Aging–Alzheimer’s Association.^32^^,^^33^ The control (Ctr) group was constituted by six healthy volunteers (3 women, 3 men, and mean age 71±9) without evidence of pathologies or of familial neurological diseases. All participants underwent a complete ophthalmological examination including the best-corrected visual acuity test. The demographic and clinical characteristics and the Mini-Mental State Exam (MMSE) scores of the recruited subjects are shown in Table 1. The study was approved by the Institutional Review Board of the University of Naples “Federico II” and all investigations adhered to the tenets of the Declaration of Helsinki (2013). Written informed consent was obtained from all the enrolled subjects.

**Table 1:** Demographic and clinical characteristics of healthy (Ctr), MCI, and AD subjects. Data expressed as mean±standard deviation. MMSE stands for minimental state examination.

	Ctr	MCI	AD
Age (years)	71±9	73±9	71±10
Women/men	3/3	3/4	7/11
Education (years)	8±5	9±5	8±5
MMSE	26.9±2.0	26.5±0.5	18.5±4.7

### 2.2 Tears Collection

Tear specimens were collected from the informed subjects and healthy volunteers. Smooth edge sterile capillary glass tubes, with internal diameter $d_{\mathrm{in}}=0.2\;\mathrm{mm}$, outside diameter $d_{\mathrm{out}}=1\;\mathrm{mm}$, and length L=12.7  mm, were used (ACCU-FILL 90 MICROPET by Becton, Dickinson and Co, Clay Adams, California, USA). During the collection, the subject’s lower eyelid was gently pulled down, and the tip of the open capillary tube was placed in contact with the tear meniscus without irritating the conjunctiva. At the least 1.0  μl tear was collected. The samples were stored at 4°C and tested by SERS within 7 days. Protein concentrations are not altered under these storage conditions.^34^

### 2.3 Surface-Enhanced Raman Spectroscopy

SERS measurements were performed with standard microscope glasses coated by a home-made colloid of gold nanoparticles (GNPs). Details of the fabrication and characterization of the GNPs are reported in Ref. 35. The average diameter of the GNP was 27 nm. About 1  μl of tears to be analyzed was dropped on the dried residual of the GNP colloid and measured a few minutes after drying. SERS signal spectra were acquired using a Jobin-Yvon instrument by Horiba Scientific ISA (Osaka, Japan) equipped with a TriAx 180 monochromator, a liquid-nitrogen cooled charge-coupled detector, and an 1800 grooves/mm grating (final spectral resolution: $2\;{\mathrm{cm}}^{-1}$). The spectra were recorded in the air at room temperature using a 17-mW-nominal power He–Ne laser source (wavelength 632.8 nm). The laser beam was focused on a $2\text{-}\mu m^{2}$ spot area of the sample surface through an Olympus microscope equipped with 100× optical objective. The laser power impinging on the sample is expected to be low enough to avoid significant heating effects, as demonstrated in preliminary measurements performed to optimize the experimental conditions. We experimentally verified that heating effects are negligible by measuring Stokes/anti-Stokes Raman response of silicon in similar acquisition conditions to the ones adopted in the present work (see, for example, Refs. 36 and 37). This is still true for tears even if their thermal conduction properties are quite different from those of silicon.^36^ All SERS spectra were acquired using an accumulation time of 180 s. At least three SERS spectra were measured in different points of the sample area and averaged. For each sample, the average signal intensity dispersion for each wavenumber point was about 8±5%. A representative spectrum for each class was obtained by averaging all SERS acquisitions from related samples.

### 2.4 Data Analysis

#### 2.4.1 Data pretreatment and deconvolution procedure

A numerical procedure based on “wavelet” algorithms was adopted to subtract the background signal and reduce the noise in the spectra.^38^ The signal was decomposed in terms of wavenumber-scaled components (wavelets), and hierarchical representation of the data was obtained. This enabled us to remove signal components due to background and noncorrelated noise through an inverse process of signal reconstruction. The data treatment was implemented using software routines (wavelet toolbox) of MATLAB^®^ (Version 7.6, MathWorks Inc., Natick, Massachusetts). In particular, “Bior6.8” biorthogonal wavelets were used. To compare the data, the spectrum intensities were scaled using the standard normal variate method, i.e., normalizing the signal to the standard deviation of intensities concerning the average signal.^39^ Some examples of SERS raw data are shown in Fig. S1 of the Supplemental Material. A best-fit numerical procedure based on the Levenberg–Marquardt nonlinear least-square method (software routines by GRAMS/AI, 2001, Thermo Electron) was used to deconvolute the spectra in terms of Lorentzian functions to determine the component modes, using as fit parameters the centers, widths, and intensities of the Lorentzian functions. One-way analysis of variance (ANOVA) was used to discriminate relevant changes in the spectra.

#### 2.4.2 PCA and i-PCA analysis

The fully processed data set of all the average spectra was analyzed by PCA and i-PCA to describe it using a set of orthogonal eigenvectors separately accounting for the various sources of the variance in the original data. PCA was performed by analyzing the covariance matrix after subtracting the mean of the variable.^40^ The fully processed average spectra data set was analyzed by PCA. The analysis was performed first on the full spectral window (400 to $1900\;{\mathrm{cm}}^{-1}$), then the spectral window was divided into 22 intervals and the PCA was performed on each spectral range (interval-PCA, i-PCA). These spectral ranges were selected by a procedure that is described in detail in Refs. 41 and 42. The first step consists of the determination of the spectral regions that feature the most significant differences among the spectra belonging to different disease classes. A reduced set of spectral “features” were selected using a statistical tool (rank features) included in the “Bioinformatics Toolbox” of MATLAB^®^ software package that selects numerically the positions and widths of the spectral regions of potential interest. Once these intervals were obtained, the ones that have specific fingerprints were selected, and the width of the intervals was optimized to give access to features assigned to DNA/RNA, lipids/carbohydrate, and proteins. The spectrum intervals obtained were: 400 to 455, 456 to 553, 554 to 706, 707 to 755, 756 to 834, 835 to 907, 908 to 969, 970 to 1031, 1032 to 1092, 1093 to 1136, 1137 to 1201, 1202 to 1307, 1308 to 1353, 1354 to 1383, 1384 to 1457, 1458 to 1529, 1530 to 1551, 1552 to 1582, 1583 to 1639, 1640 to 1712, 1713 to 1800, 1801 to $1900\;{\mathrm{cm}}^{-1}$. PCA analysis of the average spectra full data set was performed on these intervals (i-PCA). All the scores obtained by this procedure were used to build the new data set. It was inspected to outline the combination of intervals and principal component (PC) scores that maximize the differentiation of the spectra depending on their disease class. A number of PC scores and intervals were selected also taking into account the assignments of the various Raman modes outlined by the deconvolution procedure. The selected sets of i-PCAs were used to build various classification models. The classification procedure was performed using the naïve Bayes classification^43^ algorithm tool of the “Statistical Toolbox” available in MATLAB^®^ software package. The ability of the obtained classification models to recover the correct disease class of patients by using the average tears SERS spectrum was tested evaluating the overall accuracy (total number of spectra correctly classified over the total number of spectra considered) and overall misclassification rate. The aim of the procedure was to obtain a model that enabled us to correctly retrieve all the disease classes of the average spectra.

## 3 Results and Discussion

### 3.1 SERS Measurements

**Fig. 1:**
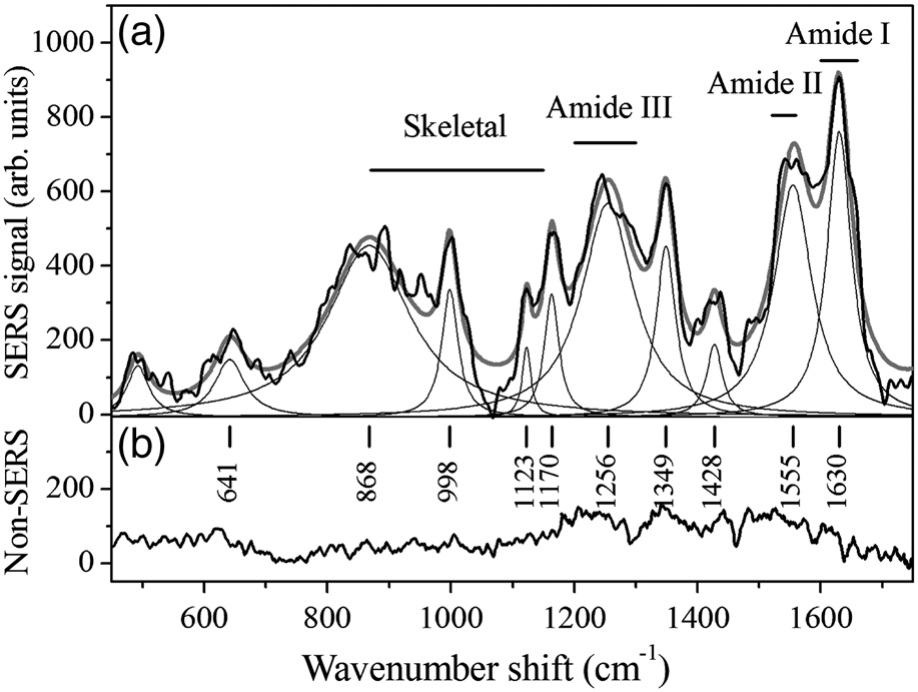
(a) SERS spectrum of tears from a healthy subject. The acquisition time was 180 s. The experimental spectrum (black curve) was fitted by a convolution of Lorentzian functions. The gray curve is the resulting fit curve. The main modes found are indicated. (b) Spectrum of tear obtained by conventional Raman spectroscopy in similar acquisition conditions.

An example of SERS response of tears of a healthy subject is shown in Fig. 1(a) along with a representative spectrum of the same subject’s tears obtained by conventional Raman spectroscopy in the same experimental conditions [Fig. 1(b)]. The Raman spectrum does not provide any valuable information apart from very faint features. Conversely, SERS spectrum clearly evidences the various contributions of tear components. The main modes indicated in the Fig. 1(a) were individuated by a deconvolution of the spectrum in terms of Lorentzians. Raman features typical of proteins are observed in the SERS spectrum shown in Fig. 1(a), where the skeletal (primary protein structure) region, the amide I, amide II, and amide III bands can be recognized. The positions of the most relevant peaks are reported in the figure, and tentative assignments of the modes identified by the deconvolution process are reported in Table 2 together with related references. In particular, three strong peaks are found at 998, 1349, and $1170\;{\mathrm{cm}}^{-1}$ and assigned to phenylalanine (Phe), tryptophan (Trp), and tyrosine (Tyr), respectively.^44^ The intense Trp mode may primarily be due to lysozyme (LZ), one of the major components of tears.^22^^,^^45^

**Table 2:** SERS of human tears: assignment of main modes according to Refs. 23 and 44 (def, deformation; wag, wagging; str, stretching; bend, bending; sciss, scissoring).

Mode position (${\mathrm{cm}}^{-1}$)	Mode	Assignment	ΔI(AD)	ΔI (MCI)
641	${\mathrm{COO}}^{-}$ wag	—	—	—
868 to 885	—	—	—	↓
998 to 1000	symm ring CC str.	Phenylalanine	↑	↓
1123	${\mathrm{NH}}_{3}^{+}$ def.	—	—	—
1163 to 1175	N-H wag	—	—	↑
1243 to 1250	β-sheet	Amide III (LF, LZ)	—	↓
1291	$\alpha \text{-}\mathrm{helix}{\mathrm{CH}}_{2}$ wag	Amide III		
1340 to 1350	C─H def	Trp; LF, LZ	—	↓
1428 to 1435	${\mathrm{CO}}^{-}$ symm. str.	LF, LZ	—	—
1459	—	—	↓	—
1516 to 1533	C─C str	Amide II	↓	—
1566	${\mathrm{NH}}_{2}$ sciss	—	—	↑
1591	—	—	↑	—
1612	Indole N-H, C═O str.	—	—	—
1630 to 1640	α-helix	Amide I	—	↑

**Fig. 2:**
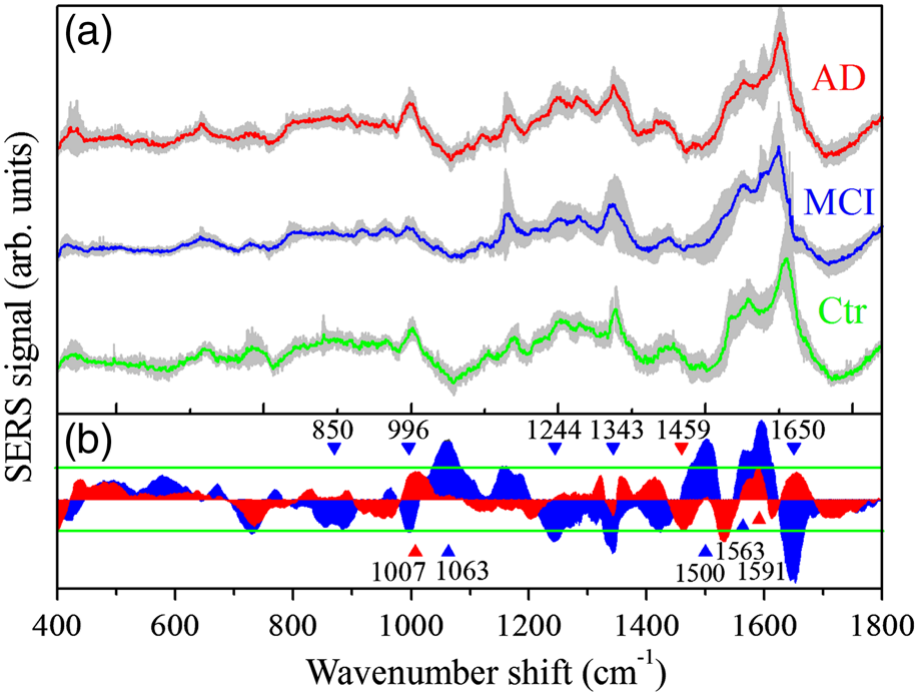
(a) Averaged SERS spectra of tear samples from healthy subjects (Ctr-green line), mild cognitive disease-affected subjects (MCI-blue line), and AD-affected subjects (AD-red line). The gray areas represent the standard deviation of the signal intensities within the considered data classes. Each of the reported spectra was obtained by averaging all SERS spectra of subjects belonging to a single class. SERS spectra were obtained by 180 s long acquisitions. The average relative dispersion of the signal intensity is 24±17% for the control set, 21±14% for the MCI data, and 37±8% for the AD data, respectively. (b) Signal differences concerning the control data of AD (red area) and MCI (blue area) spectrum. The green lines indicate the signal dispersion range (0.68 of the standard deviation). The statistically significant signal differences (p-value <0.05 in the one-way ANOVA statistics) are indicated by blue (MCI) and red (AD) marks, respectively.

SERS peaks at about 1256 and $1428\;{\mathrm{cm}}^{-1}$ are assigned to LZ or lactoferrin (LF) in agreement with conventional Raman spectroscopy results for single-component solutions of LF and LZ,^22^^,^^45^ and with SERS response of LZ.^45^ These tentative assignments are in agreement with previous studies showing that the major components of tears are immunoglobulins (IgA), lactoferrin, lysozyme, lipocalin, and albumin.^17^^,^^46^ Among these components, lactoferrin and lysozyme have an important role in the eye functionality; indeed they provide defense mechanisms against infective agents.^47^ Figure 2 shows the average SERS spectra for the three classes of subjects here considered (Ctr for control/healthy subjects; MCI for subjects suffering from MCI; AD subjects suffering from AD). The average relative dispersion of the signal intensity is 24±17% for the control set, 21±14% for the MCI data, and 37±8% for the AD data. The three spectra in Fig. 2(a) share some common features even if differences can be noticed. Spectra obtained by subtracting the control spectrum from the MCI and AD signals are reported in Fig. 2(b). In some spectral regions, the intensity variation is larger than the data dispersion. In particular, statistical deviations were determined by one-way ANOVA test and are indicated in Fig. 2(b) for AD and MCI signals. Positive deviations are found at 1007 and $1591\;{\mathrm{cm}}^{-1}$ and a negative deviation at $1459\;{\mathrm{cm}}^{-1}$ for the data related to the AD class. A positive deviations at $1563\;{\mathrm{cm}}^{-1}$ and negative deviations at 850 to 885, 996, 1244, 1343, and $1650\;{\mathrm{cm}}^{-1}$ are determined for the data of MCI class. Two large intensity deviations of MCI signal occur at 1063 and $1500\;{\mathrm{cm}}^{-1}$ but they were be not further considered being close to minima of the SERS spectrum.

The occurrence of an intensity increase or decrease of the SERS signal of AD and MCI samples with respect to the Ctr sample-related spectrum is indicated in Table 2 by ↑ and ↓ symbols, respectively. It is noteworthy to note that SERS spectra from MCI-affected subjects exhibits a signal intensity lower than the reference spectrum (from healthy subjects) for modes at 1343 and $1243\;{\mathrm{cm}}^{-1}$. These modes are assigned to the C-H deformation and amide III β-sheet, respectively.^24^ Their intensity change indicates a modification of protein secondary structures that can result from altered physiological conditions. Kim et al.^24^ reported a correlation between the $I_{1342}/I_{1243}$ ratio of SERS modes at 1243 and $1342\;{\mathrm{cm}}^{-1}$ and eye disease state in human tears. They found an increase of the $I_{1342}/I_{1243}$ ratio in subjects affected by adenovirus ($I_{1342}/I_{1243}=1.13$) and herpes simplex ($I_{1342}/I_{1243}=3.73$) versus a control value $I_{1342}/I_{1243}=0.8$. In the present case, the mean value estimated for $I_{1342}/I_{1243}$ is 1.1±0.4 for healthy subjects, 1.5±0.7 for samples from AD class, and 1.8±0.7 for tear samples from MCI class. These average ratio values are larger in AD and MCI cases than in control; however, the data dispersion is too large to find a significant correlation.

**Fig. 3:**
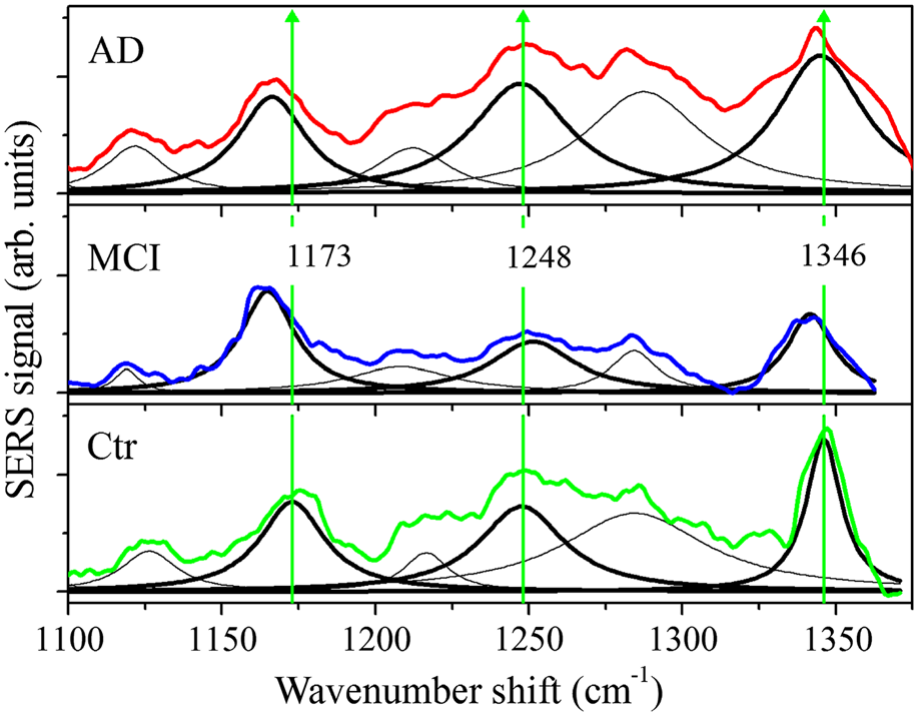
Comparison of averaged SERS spectra of tears from AD-affected (red curve), MCI-affected (blue curve), and Ctr healthy (green curve) subjects in the amide III region. All SERS spectra were obtained by 180 s long acquisitions. The mode peaks resulting from the fitting spectrum deconvolution are reported together with the experimental data. Thick curves indicate selected modes featuring larger differences with respect to the control spectrum (see text).

**Fig. 4:**
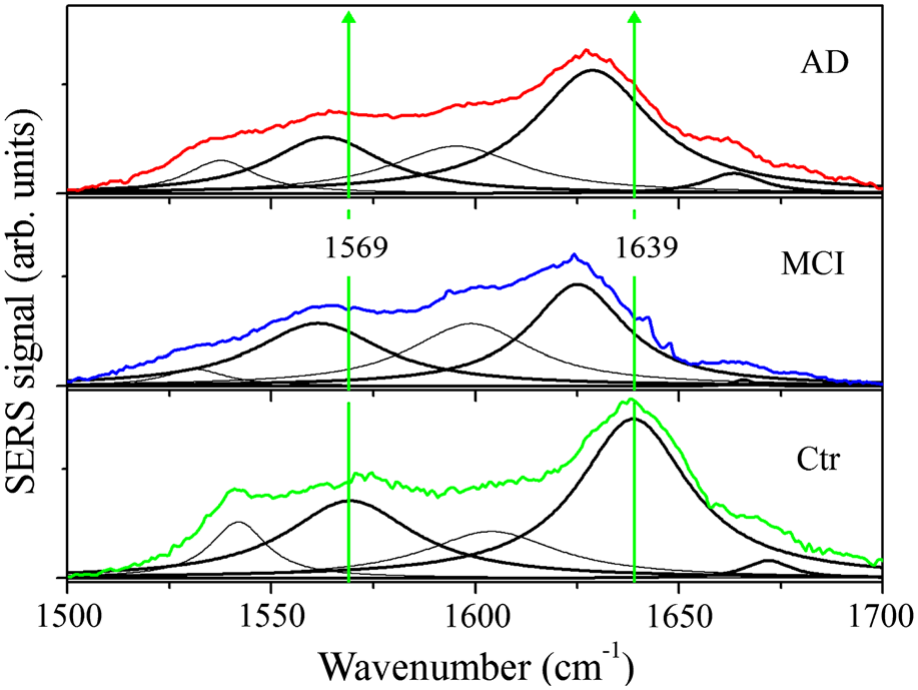
Comparison of averaged SERS spectra of tears from AD-affected (red curve), MCI-affected (blue curve), and Ctr healthy (green curve) subjects in the 1500 to $1700\;{\mathrm{cm}}^{-1}$ range including amide II and amide I bands. All SERS spectra were obtained by 180 s long acquisitions. The mode peaks resulting from the fitting spectrum deconvolution are reported together with the experimental data. Thick curves indicate selected modes featuring larger differences with respect to the control spectrum (see text).

Figure 3 shows a comparison between the average SERS spectra of healthy (Ctr), MCI- and AD-affected subjects for the amide III (1100 to $1400\;{\mathrm{cm}}^{-1}$) region together with their deconvolution in terms of Lorentzian functions. The three main components in the Ctr spectrum occur at 1173, 1248, and $1346\;{\mathrm{cm}}^{-1}$. These modes are compared with the equivalent components of MCI and AD spectra. The mode found at $1173\;{\mathrm{cm}}^{-1}$ in the Ctr spectrum and assigned to tyrosine is centered at a lower wavenumber value (about $1165\;{\mathrm{cm}}^{-1}$) in the MCI and AD spectra. The level of tyrosine is expected to decrease in intensity during the process of amyloid fibril formation,^48^ and the observed weakening of the mode energy indicates a degradation of protein structure. The average SERS intensity of the MCI spectrum in the 1244 to $1350\;{\mathrm{cm}}^{-1}$ range is lower than the intensity in the other two cases. The AD spectrum deconvolution is characterized by relatively broader modes, which indicate an increased disorder of the protein secondary structure. A shift to lower energies of SERS modes is also observed in the MCI and AD spectra of Fig. 4, in which the amide I and amide II band regions are reported with the result of the signal deconvolution in terms of Lorentzians. The amide I band of the spectrum (Ctr) from healthy subjects is centered at $1639\;{\mathrm{cm}}^{-1}$. This band is shifted to 1625 and $1628\;{\mathrm{cm}}^{-1}$ in MCI and AD cases, respectively. These changes could be due to an increase contribution of the random coil component and a decrease of the intensity of the α-helix-related mode. The overall effect of the combination of these two intensity changes is to move the center of the amide I band toward lower wavenumber shift values. It is noteworthy that also hydration causes a faint change of α-helix contribution as reported in a Raman study on lysozyme.^49^ It is interesting to note that the intensity of the amide I band is lower than the control data only in the MCI case. All the observed changes suggest a modification of the secondary structure of the proteins stronger in MCI samples than in AD samples. Notwithstanding the evidence that the changes in the tear composition induced by different physiological conditions influence their SERS response, it is hard to obtain a clear indication on the occurrence of AD and MCI diseases due to the presence in the samples of confounder proteins having strong spectral similarities with disease markers as amyloids and tau protein.^50^

### 3.2 Patient Clinical State Recall Using i-PCA Results

SERS spectra of tears reported herein are very complex as expected for these body fluids. In addition, the differences among SERS spectra of tears from subjects with different clinical states are sometimes very subtle and are mainly localized in some specific spectral ranges. Notably, we found that PCA performed on the whole spectral range did not differentiate the subtle changes that occurred in the examined spectra. To better evidence these differences and to profit from them to classify the spectra, we performed the PCA in the intervals reported in Sec. 2.4.2 (i-PCA). The scores of the first seven PCs obtained for all the intervals were examined. The score plots for the single intervals do not suggest simple grouping related to the state of dementia. As examples, the i-PCA results for some of the intervals are reported in the Supplemental Material in terms of score plots and loadings of the first two components (see Fig. S2 in the Supplemental Material). This finding suggested the need for a complex model for classification. Accordingly, the score coefficients obtained by i-PCA are used to build models enabling them to obtain the correct classification of the spectrum to one of the dementia disease classes of the corresponding subject. The classification models were built using the naïve Bayes classification algorithm,^43^ according to the procedure described in Sec. 2.4. The ability of the various models to obtain the correct classification of the average spectrum to one of the disease classes was barely evaluated by considering the overall accuracy in order to prove that the model itself is able to recall the correct disease class for all the considered spectra. To obtain a 100% accuracy in recalling the disease class of the spectra dataset used to build the model by using the SERS spectra of tears, we built a model using the scores of PC3, PC4, PC5, and PC6 calculated in the intervals 456 to 553, 707 to 755, 1202 to 1307, 1308 to 1353, 1354 to 1383, 1552 to 1582, 1583 to $1639\;{\mathrm{cm}}^{-1}$, which enabled us to obtain the correct recall of all the spectra (i.e., 100% of overall accuracy and 0% of overall misclassification rate) (representative outcomes of the i-PCA in terms of score higher-order score components are reported in Fig. S3 in the Supplemental Material). Since the problem is a multiclass classification (i.e., a classification procedure involving more than two classes) (see Ref. 51 and references therein) because three clinical states are considered and the dataset is small and unbalanced (6, 7, and 18 patients belong to the Ctr, MCI-affected, and AD-affected disease classes, respectively), it was not possible to perform further studies using standard validation procedures for the model. Nevertheless, these results are encouraging since they confirm the ability of correctly recovering the clinical state of patients by analyzing tears SERS spectra, thus offering proof of the potential of the method that could be usefully applied for classifying spectra acquired from new enrolled patients. These results boost further studies involving more and properly selected patients, and SERS spectra that will allow to test the efficacy of the use of different intervals for i-PCA, and/or the use of different classification methods including neural network algorithms.

## 4 Conclusions

Optical spectroscopy methods can provide a unique way for analyzing body fluids, enabling fast, sensitive, noninvasive, and reliable diagnosis, as well as large-scale medical screening campaigns. We analyzed SERS response of tears, aiming to evince differences in the spectra acquired from tears collected from AD subjects compared to samples from healthy subjects and subjects with ascertained mild-cognitive symptoms. Differences were found in various spectral regions. In particular, the spectral region related to protein components showed the largest changes indicating conformational changes that can be mainly ascribed to lactoferrin and lysozyme. In fact, as aforementioned, these substances are among the principal tear components. In addition, the determination of $I_{1342}/I_{1243}$ ratio allowed us to obtain quantitative information on changes related to pathological states, similarly to those reported for inflammatory alterations. Even if it was not possible to discriminate specific biomarkers of AD, the global SERS response reflects small but interesting modifications of the tear composition that can be attributed to altered levels of specific pathological and disease-stimulated substances. The interval partial component analysis (i-PCA) of the spectra allowed us to discriminate AD-affected subjects from healthy and MCI-affected subjects. The classification accuracy of the built method was very encouraging with interesting perspectives for medical applications as support of clinical diagnosis and discrimination of AD from other forms of dementia. Thus, the proposed method provides useful information regarding the diagnostic classification of subjects with cognitive impairment and deserves attention and an additional systematic study on a large number of subjects.
