# Supplementary material for: Surface-enhanced Raman spectroscopy of tears: toward a diagnostic tool for neurodegenerative disease identification
Source: J Biomed Opt. 2020 Aug 6;25(8):087002. doi: 10.1117/1.JBO.25.8.087002 (PMC7406892; doi:10.1117/1.JBO.25.8.087002)
Supplement: Supplementary file 1 [file JBO_025_087002_SD003.doc]

Surface-enhanced Raman spectroscopy of tears: towards a diagnostic tool for neurodegenerative disease identification

Supplementary Material

**G. Cennamo^a^, D. Montorio^b^, V. Brescia Morra^b^, C. Criscuolo^b^, R. Lanzillo^b^, E. Salvatore^b^, C. Camerlingo^c^*, M. Lisitskiy^c^, I. Delfino^d^, M. Portaccio^e^, M. Lepore^e^**

**^a^ Dipartimento di Sanità Pubblica, Università "Federico II" di Napoli, 80100 Napoli, Italy.**

**^b^ Dipartimento di Neuroscienze e Scienze Riproduttive e Odostomatologiche, Università "Federico II" di Napoli, 80100 Napoli, Italy.**

**^c^ CNR-SPIN, Istituto Superconduttori, Materiali Innovativi e Dispositivi, Consiglio Nazionale delle Ricerche, 80078 Pozzuoli, Italy.**

**^d^ Dipartimento di Scienze Ecologiche e Biologiche,Università della Tuscia, 01100 Viterbo, Italy.**

**^e^ Dipartimento di Medicina Sperimentale, Università della Campania "L. Vanvitelli", 80100 Napoli, Italy.**

***Supplementary results***

***SERS data***

Examples of SERS raw data of tears from a control subject (Ctr), a mild cognitive impairment (MCI), and an Alzheimer’s disease affected (AD) subject are reported in Fig. S1 (a), (b), and (c) respectively. The curves refer to spectra acquired in different points of the tear samples. For each spectrum, the background signal was evaluated by the wavelet-based algorithm and represented by the dashed curves. After the subtraction of the background signal, the spectra were normalized using vector normalization method. The resulting spectra were reported in Fig. S1 (d), (e), and (f) for the Ctr, MCI and AD cases, respectively.

| 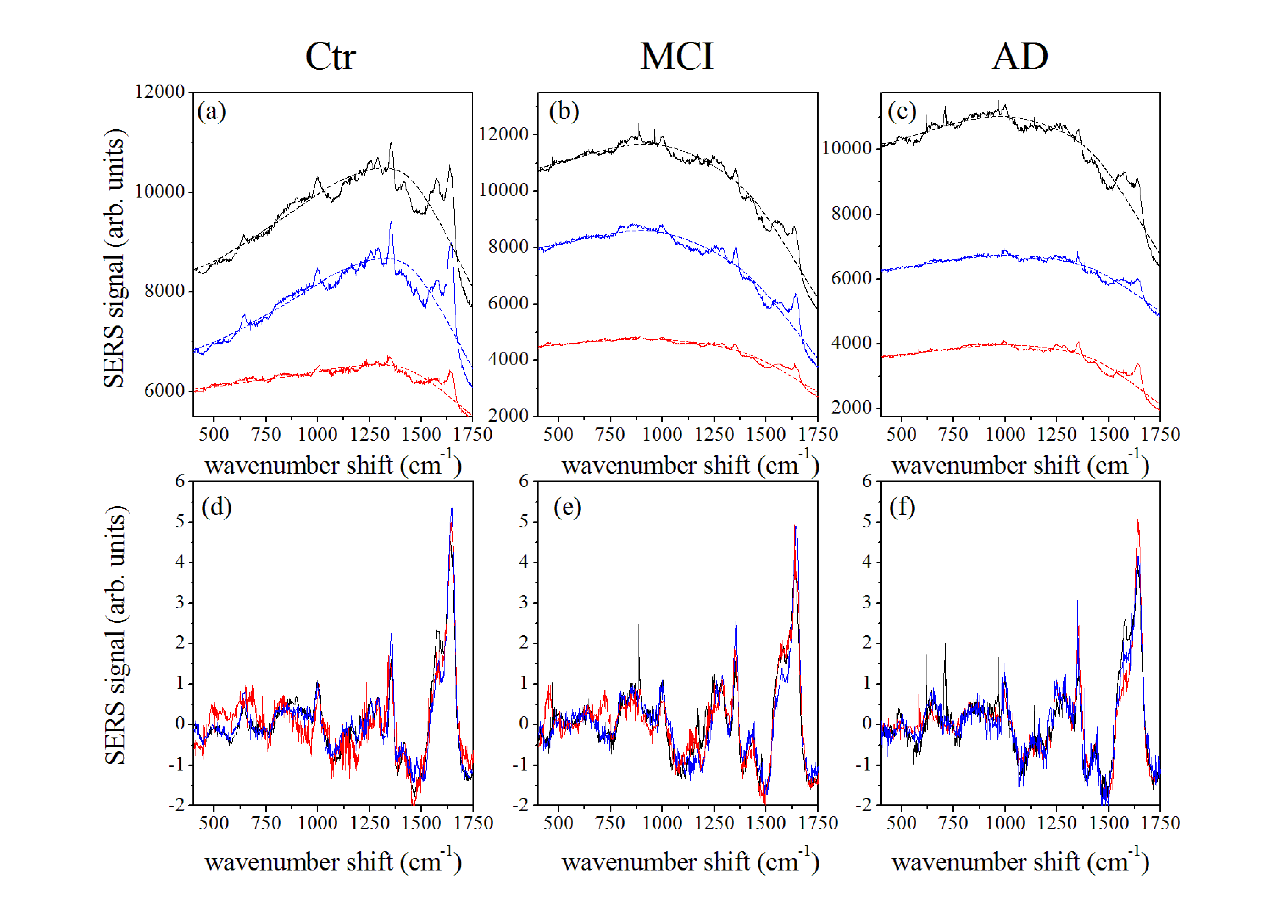 |
| --- |
| **Fig. S1** Raw data of SERS spectra of tears from (a) a Ctr, (b) a MCI, and (c) an AD subject. The background signals of SERS signal were estimated by a wavelet-based numerical algorithm and were reported by the dashed curves. After the subtraction of the background signal, the spectra were normalized using vector normalization method. The resulting spectra were reported in (d), (e), and (f) for the Ctr, MCI and AD cases, respectively. |

## **i-PCA results**

Representative outcomes of the i-PCA performed on defined intervals are reported in Fig. S2 in terms of score plots and loadings for the first two principal components. In particular, results obtained for the 707-755, 970-1018, 1202-1307, 1583-1639 cm^-1^ are shown.

| **** |
| --- |
| **Fig. S2** Results of i-PCA on the whole dataset (average spectra obtained for all the patients). PC2 vs PC1 score plot representation of the data for healthy/Ctr (green triangles), MCI-affected (blue triangles) and AD-affected (red squares) subjects for the following spectral ranges (intervals): **(a)** 707-755 cm^-1^, **(c)** 970-1018 cm^-1^, **(e)** 1202-1307 cm^-1^, **(g)** 1583-1639 cm^-1^. The corresponding loadings of the first two components in the same intervals are reported in **(b)** 707-755 cm^-1^ , **(d)** 970-1018 cm^-1^, **(f)** 1202-1307 cm^-1^, **(h)** 1583-1639 cm^-1^ ranges. |

Representative outcomes of the i-PCA in terms of score plots are reported in Fig. S3. Higher-order score components are considered, namely PC3, PC4 and PC5 for the 1202-1307 cm^-1^ range (12^th^ interval, Figs. S3 (**a)** and S3 (**b**)) and PC2, PC3 and PC4 for the 1583-1639 cm^-1^ range (19^th^ interval, Figs. S3 (**c)** and S3 (**d)**). Projections in the PC3-PC4 plane are considered for PC5 < 1×10^-4^  scores in Fig. S2 (**a)** and for PC5 > 1×10^-4^ in Fig. S2 (**b)**, respectively. Similarly, in Figs. S3 (**c)** are reported the projections in the PC2-PC3 plane for PC4 < -4×10^-4^ and PC4 >1×10^-4^ score values and in S3 (**d**) for -4×10^-4^ < PC4< 1×10^-4^ values, respectively.

|  |
| --- |
| **Fig. S3** Results of i-PCA on the whole dataset (average spectra obtained for all the patients). High-order components score plot representation of the data for healthy/Ctr (green triangles), MCI-affected (blue triangles) and AD-affected (red squares) subjects for the **(a-b)** 1302-1307 cm^-1^ and **(c-d)** 1583-1639 cm^-1^ spectral ranges (12^th^ and 19^th^ intervals). In (**a-b**) panels score values in the PC3-PC4 plane are reported for PC5< 1×10^-4^ and PC5> 1×10^-4^, respectively. In (**c-d**) panels score values in the PC2-PC3 plane are reported for PC4 < -4×10^-4^ or PC4> 1×10^-4^, respectively. |
